# Comparison of three techniques in the surgical management of metastatic vertebral fracture with posterior wall damage: a retrospective study

**DOI:** 10.1186/s13018-023-03608-9

**Published:** 2023-02-23

**Authors:** Heng Wang, Jile Xie, Yijie Liu, Guangdong Chen, Weimin Jiang

**Affiliations:** 1 Department of Orthopaedic Surgery, First Affiliated Hospital of Soochow University, 899 Pinghai Road, Suzhou, China; 2 Department of Orthopaedic Surgery, Dushu Lake Hospital Affiliated to Soochow University, 9 Chongwen Road, Suzhou, China

**Keywords:** Kyphoplasty, Spinal metastases, Spine, Vertebral fracture, Pedicle screw fixation

## Abstract

**Background:**

To retrospectively compare the safety and efficacy of percutaneous kyphoplasty (PKP), internal fixation (IF), and kyphoplasty combined with internal fixation (KP + IF) in treating metastatic vertebral fracture (MVF) with posterior wall damage.

**Methods:**

87 patients with MVF with posterior wall damage underwent surgery. In Group PKP, 36 patients underwent PKP; in Group IF, 20 patients underwent pedicle screw fixation; and in Group KP + IF, 31 patients underwent kyphoplasty combined with pedicle screw fixation. Operative time, intraoperative blood loss, clinical and radiological results, and complication rate in each group were evaluated and compared.

**Results:**

Significant improvement on the VAS, ODI scores, vertebral height and local kyphotic angle (LKA) was noted in each group (*P* < 0.001). Group PKP and Group KP + IF achieved better pain relief than Group IF (*P* < 0.05). At postoperative 3 days, Group PKP had better pain relief than Group KP + IF (*P* < 0.05). At other follow-up time points, there were no differences between Group PKP and KP + IF (*P* > 0.05). Group KP + IF and Group IF were more efficacious than Group PKP in terms of height restoration and LKA correction (*P* < 0.05). Group KP + IF had a higher incidence of postoperative complications than Group PKP and Group IF(*P* < 0.05).

**Conclusions:**

PKP was safe and effective in treating MVF with posterior wall damage. It can achieve similar clinical outcomes compared to KP + IF, but associated with less operative time, less blood loss and fewer complications. IF alone should not be the first treatment option for its poorer analgesic effect.

## Background

The spine is the most common site of osseous metastases, and 33% to 70% of cancer patients developed spinal metastases during their clinical course [1, 2]. The affected individuals always present as severe back pain when metastatic vertebral fracture (MVF) occurs. Conservative treatments, which include analgesics, radiotherapy, chemotherapy, hormone therapy and bisphosphonates, are rarely effective or short-acting in relieving pain and improving ambulatory status. Open surgeries are often associated with high invasiveness and complication rate. Currently, percutaneous kyphoplasty (PKP) has been widely accepted as an effective treatment option for metastatic vertebral fracture (MVF), and many encouraging studies have been reported [3–5].

However, PKP is considered relatively or even absolutely contraindicated in the treatment of vertebral fracture with posterior wall damage [6–9]. There have been safety concerns over high risk of cement leaking posteriorly and further tumor retropulsion into spinal canal, which may result in neural injury. Therefore, surgeons are more inclined to perform open surgeries including internal fixation (IF) alone or kyphoplasty (KP) combined with IF in such cases. Only few studies reported on the role of PKP in the treatment of vertebral fracture with posterior wall damage related to osteoporosis or cancer [10, 11]. But it is still not clear whether PKP alone could provide satisfactory efficacy and safety compared with IF or KP combined with IF in these patients. To address this issue, this study evaluated and compared the safety and clinical efficacy, especially the pain reduction, of PKP, IF, and KP combined with IF in treating MVF with posterior wall damage.

## Materials and methods

### Study population

This study included 87 patients with a single-level thoracolumbar (Th5–L5) MVF with posterior wall damage who underwent surgery in our spine surgery center between January 2010 and December 2019. This retrospective clinical study was approved by the Institutional Ethics Committee of Soochow University, and informed consent was obtained from all patients.

The inclusion criteria were: (1) a definitive diagnosis of MVF, which was confirmed by a multidisciplinary team including experienced radiologist, orthopedic surgeon and oncologist, (2) the pain region was consistent with radiological findings, (3) severe back pain refractory to conservative therapy, and (4) survival time > 3 months.

The exclusion criteria were: (1) patients with neurological deficits, (2) previous spinal surgery, (3) spinal cord compression on magnetic resonance imaging (MRI), and (4) severe medical comorbidities (e.g., heart failure).

For patients who developed new spinal metastases and underwent multiple-round surgeries, only the first round was analyzed. All patients were informed the advantages and disadvantages of different surgeries and chose the method as their own wishes. According to the surgical technique, the 87 patients were divided into 3 groups: Group PKP, who underwent PKP alone; Group IF, who underwent pedicle screw fixation; and Group KP + IF, who underwent KP combined with pedicle screw fixation and prophylactic decompression (Table 1). Group PKP consisted of 36 patients (16 men and 20 women), aged 45–75 years (average, 61.1 years old). The treated levels included 14 thoracic vertebras and 22 lumbar vertebras. Group IF consisted of 20 patients (8 men and 12 women), aged 40–66 years (average, 54.4 years old). The treated levels included 8 thoracic vertebras and 12 lumbar vertebras. Group KP + IF consisted of 31 patients (15 men and 16 women), aged 40–62 years (average, 52.8 years old). The treated levels included 18 thoracic vertebras and 13 lumbar vertebras.

**Table 1:** Demographic data of patients

	Group PKP	Group IF	Group KP + IF
Patients, no	36	20	31
Sex (men:women)	16:20	8:12	15:16
Age (years)	61.1 ± 12.2	54.4 ± 9.1	52.8 ± 7.5
Treated levels, no	Thoracic (14); lumbar (22)	Thoracic (8); lumbar (12)	Thoracic (18); lumbar (13)
Primary malignancy			
Breast cancer	11	7	12
Lung cancer	7	4	7
Prostate cancer	6	4	3
Liver cancer	2	1	2
Kidney cancer	2	2	1
Thyroid cancer	1	0	1
Colon cancer	1	0	1
Stomach cancer	3	1	1
Esophagus cancer	1	0	2
Nasopharyngeal cancer	1	0	1
Cervical cancer	1	1	0

### Preoperative preparation

The patients’ diagnosis was confirmed by a multidisciplinary team including experienced orthopedic surgeons, radiologists, and oncologists. The patients’ general condition was comprehensively evaluated. All patients were examined by X-ray and computerized tomography (CT) scan to evaluate the fracture configuration and vertebral wall integrity. MRI was also performed to evaluate the spinal canal compromise.

### Surgical procedure

All the procedures were performed with the patients in prone position under general anesthesia.

**Fig. 1:**
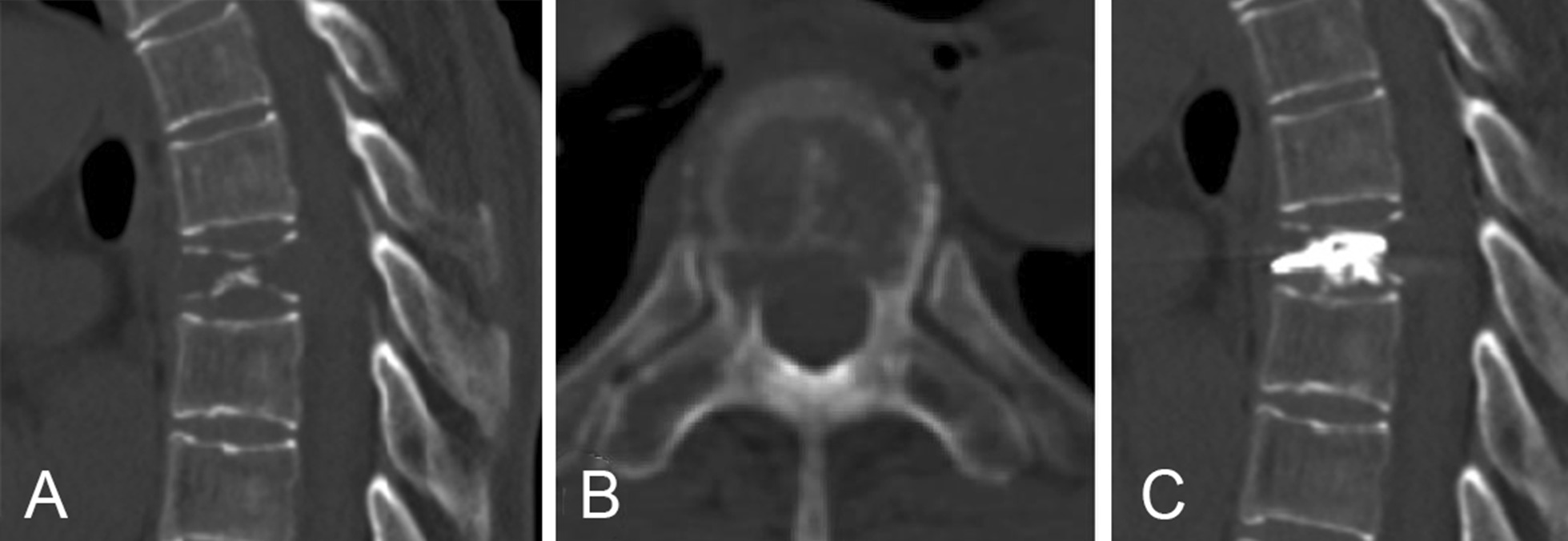
Preoperative CT in an adult patient who had metastatic vertebral fracture with posterior wall damage (**A**, **B**); she underwent percutaneous kyphoplasty (**C**)

Group PKP (Fig. 1): The operative technique for PKP has been well described [12, 13]. Biopsy was routinely performed after the bilateral working channels were established. The balloons were inflated slowly and the inflation was stopped when the pressure reached 200 psi or the balloon contacted the endplate. Then, the balloons were deflated and removed, followed by polymethylmethacrylate (PMMA) cement injection. Injection was stopped immediately if high resistance was encountered or if PMMA neared the posterior wall of the vertebral body.

**Fig. 2:**
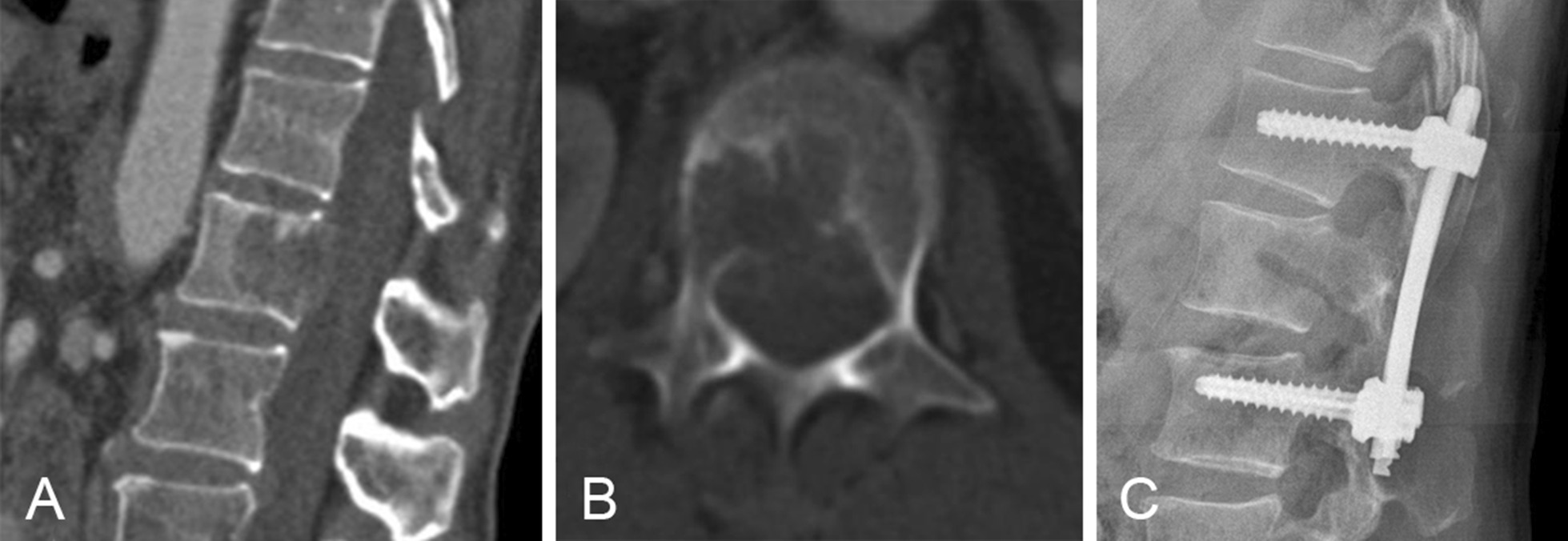
Preoperative CT in an adult patient who had metastatic vertebral fracture with posterior wall damage (**A**, **B**); she underwent pedicle screw fixation (**C**)

Group IF (Fig. 2): Four pedicle screws were placed into the segments above and below the metastatic vertebra. For the affected vertebra, bilateral transpedicular puncture and biopsy were performed.

**Fig. 3:**
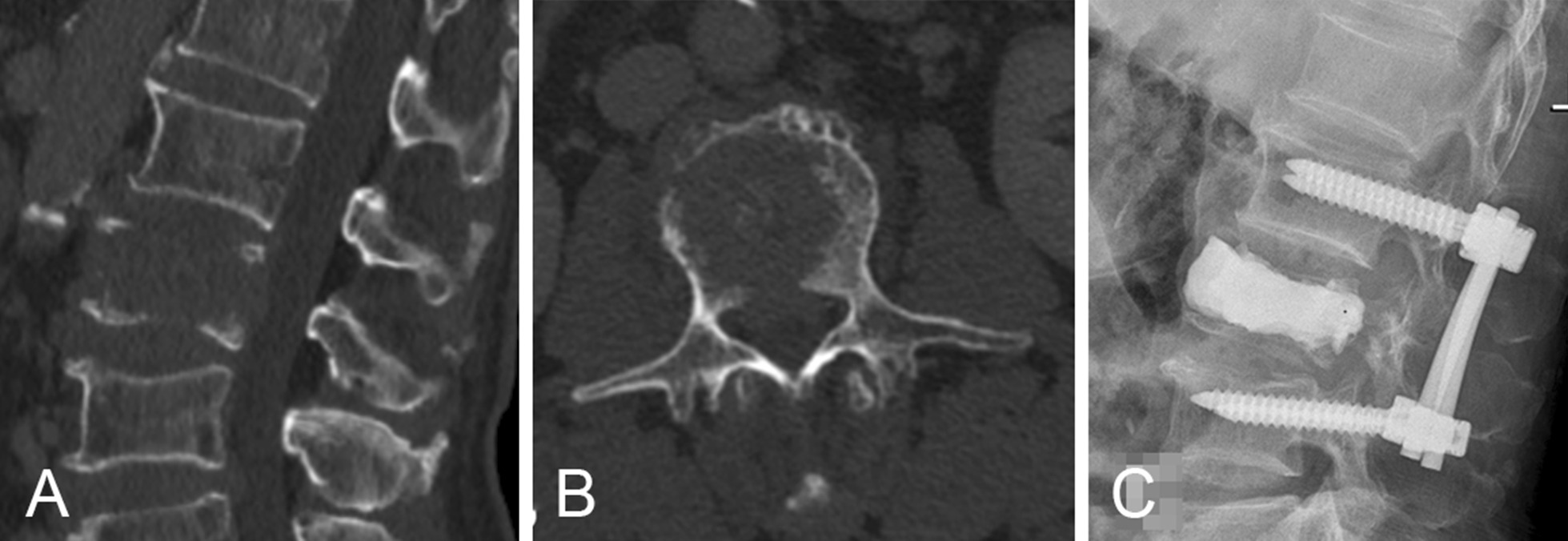
Preoperative CT in an adult patient who had metastatic vertebral fracture with posterior wall damage (**A**, **B**); he underwent kyphoplasty combined with pedicle screw fixation (**C**)

Group KP + IF (Fig. 3): Four pedicle screws were placed into the adjacent vertebral bodies and then propped up properly to restore the vertebral body height. Then, biopsy and kyphoplasty were performed in the metastatic vertebra, and laminectomy was performed for prophylactic decompression.

### Postoperative treatment

The patients’ vital signs and neurological status were monitored for 12 h postoperatively. Antibiotics were used for 24–48 h, and analgesic measures were given as needed. Patients were allowed to stand and walk 1–3 days postoperatively wearing a thoracolumbar brace. All patients received oncology adjuvant treatments based on the primary malignancy.

### Data collection and outcome evaluation

Clinical and radiological data were recorded and evaluated at pre-operation, and at 3 days, 1 month, 6 months, and 12 months after surgery.

The clinical effects of patients were evaluated using the visual analog scale (VAS; scored from 0 to 10: 0, no pain; 10, the worst imagined) and Oswestry Disability Index (ODI) score. The anterior vertebral heights (AVH), middle vertebral heights (MVH) and local kyphotic angle (LKA) in Cobb method of the affected vertebra were measured on lateral radiographs.

### Statistical analysis

The operation time and blood loss of each group were analyzed with one-way ANOVA test. The clinical and radiological outcomes in each group before and after surgeries were analyzed by two-way ANOVA with Tukey’s multiple comparisons. The complication rate of each group was analyzed by Chi-square test (Fisher’s exact test). All the statistical analysis was performed by GraphPad Prism 7.0 software (USA). The difference was considered to be statistically significant at the *P* < 0.05.

## Results

All 87 patients tolerated the operation well. No neurological injury caused by further tumor retropulsion was found. The average operation time in PKP, IF, and KP + IF groups was 49.9 ± 9.3, 86.2 ± 8.4, and 155.9 ± 22.9 min, respectively. The average blood loss was 15.0 ± 7.7, 87.5 ± 27.2, and 221.9 ± 62.8 mL, respectively. The results were significantly different among the 3 groups (*P* < 0.001) (Table 2).

**Table 2:** Comparisons of clinical and radiological results between groups

	Group PKP VS Group IF	Group IF VS Group KP + IF	Group PKP VS Group KP + IF
Age	*P* = 0.007	*P* = 0.749	*P* < 0.0001
Sex	*P* = 0.785	*P* = 0.580	*P* = 0.809
Operation time	*P* < 0.001	*P* < 0.001	*P* < 0.001
Blood loss	*P* < 0.001	*P* < 0.001	*P* < 0.001
VAS			
Pre	*P* = 0.548	*P* = 0.933	*P* = 0.719
Postoperative 3 days	*P* < 0.001	*P* = 0.028	*P* = 0.022
Postoperative 1 month	*P* = 0.023	*P* = 0.029	*P* > 0.999
Postoperative 6 months	*P* = 0.046	*P* = 0.010	*P* = 0.759
Postoperative 12 months	*P* = 0.029	*P* = 0.012	*P* = 0.899
Complications	*P* > 0.999	*P* = 0.053	*P* = 0.021

During the 12 months follow-up, 11 patients developed new spinal metastases and 12 patients died (Table 3). Most of the patients (9/11) who developed new spinal metastases chose to receive PKP.

**Table 3:** Patients number during follow-up period

	Group PKP	Group IF	Group KP + IF
Postoperative 6 months			
Patients, no	30	18	26
Excluded cases, no	6 (3 developed new spinal metastases; 3 died)	2 (died)	5 (3 developed new spinal metastases; 2 died)
Postoperative 12 months			
Patients, no	26	16	22
Excluded cases, no	4 (2 developed new spinal metastases; 2 died)	2 (1 developed new spinal metastases; 1 died)	4 (2 developed new spinal metastases; 2 died)
Postoperative 24 months			
Patients, no	20	13	16
Excluded cases, no	6 (2 developed new spinal metastases; 4 died)	3 (1 developed new spinal metastases; 2 died)	6 (2 developed new spinal metastases; 4 died)

### Clinical outcome

**Fig. 4:**
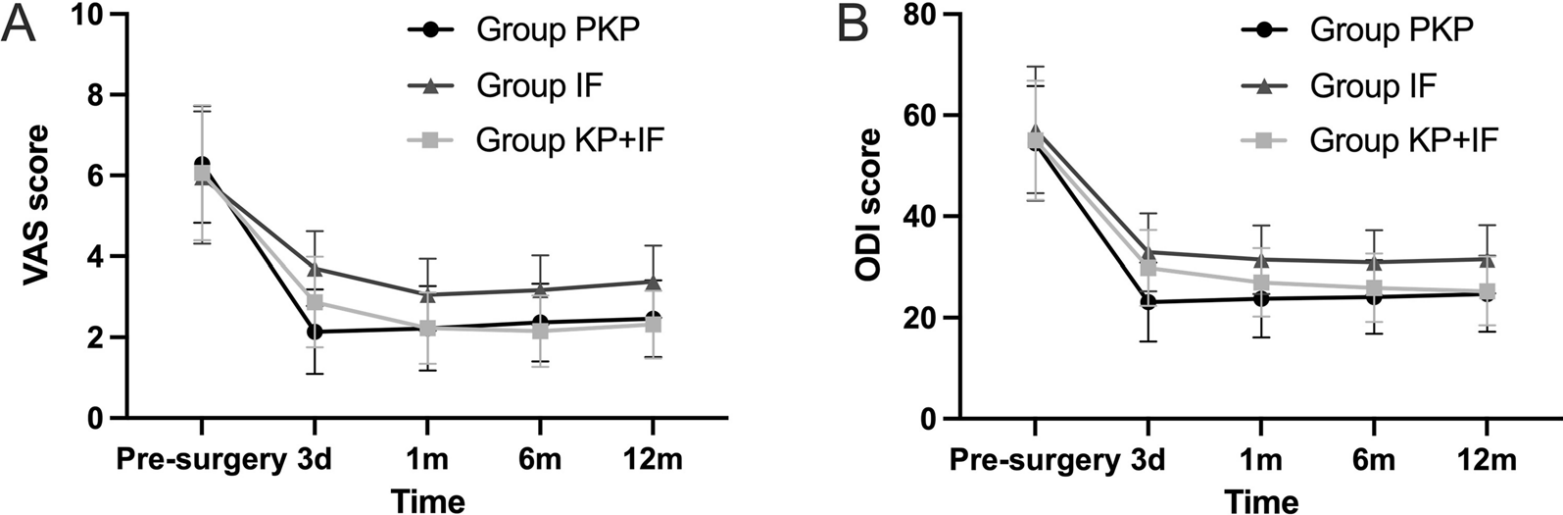
Line graphs showing comparison of clinical results among 3 groups. Change trend of the VAS score (**A**); Change trend of the ODI score (**B**)

All patients achieved significant pain relief after surgery. The VAS score decreased from 6.3 ± 1.4 to 2.1 ± 1.0 in Group PKP (*P* < 0.05), from 6.0 ± 1.6 to 3.7 ± 0.9 in Group IF (*P* < 0.05), and 6.1 ± 1.7 to 2.9 ± 1.1 in Group KP + IF (*P* < 0.05), respectively (Fig. 4). The ODI score decreased from 54.4 ± 11.4 to 23.1 ± 7.8 in Group PKP (*P* < 0.05), from 57.1 ± 12.6 to 32.9 ± 7.7.4 in Group IF (*P* < 0.05), and 55.0 ± 11.8 to 29.8 ± 7.5 in Group KP + IF (*P* < 0.05), respectively (Fig. 4).

As to pain reduction, multiple comparisons among the 3 groups revealed statistical differences (Table 2). Group PKP and Group KP + IF had better pain relief than Group IF at all follow-up time points (*P* < 0.05). At postoperative 3 days, Group PKP had better pain relief than Group KP + IF (*P* < 0.05). At other follow-up time points, there was no difference between Group PKP and KP + IF (*P* > 0.05, Table 2).

### Radiological outcome

Significant increases in the anterior and middle vertebral heights were observed after surgery in all 3 groups, and the vertebral heights were maintained throughout the follow-up. The mean improvement in LKA was 5.0 ± 2.2°, 10.6 ± 6.4°, and 10.0 ± 6.7°in Group PKP, Group IF, and Group KP + IF, respectively, and the correction was maintained at the final follow-up in all groups (Table 4). Group KP + IF and Group IF were more efficacious than Group PKP in terms of height restoration and LKA correction (*P* < 0.05).

**Table 4:** Radiological data evaluated before surgery and during follow-up

	Group PKP	Group IF	Group KP + IF
AVH			
Preoperative	51.8 ± 13.8	57.4 ± 20.7	57.3 ± 20.3
Postoperative 3 days	64.1 ± 13.7	80.4 ± 10.0	84.1 ± 8.2
Postoperative 12 months	62.9 ± 13.4	76.6 ± 9.8	79.0 ± 8.5
MVH			
Preoperative	63.0 ± 9.4	63.6 ± 17.3	64.6 ± 16.6
Postoperative 3 days	74.6 ± 9.0	84.5 ± 8.6	87.3 ± 6.3
Postoperative 12 months	72.1 ± 9.9	82.8 ± 9.5	84.2 ± 6.4
LKA (°)			
Preoperative	12.7 ± 4.3	15.2 ± 7.6	15.0 ± 7.3
Postoperative 3 days	7.8 ± 3.5 (5.0 ± 2.2)	4.6 ± 2.1 (10.6 ± 6.4)	5.0 ± 2.8 (10.0 ± 6.7)
Postoperative 12 months	8.3 ± 3.7	4.9 ± 2.0	5.2 ± 3.2

### Complications

In the Group PKP, 4 patients (4/36) had asymptomatic cement leakage with 2 cases into the intervertebral space, 1 case lateral to the vertebral body and 1 case into the paravertebral vein. In the Group IF, 2 patients (10%) had complications including local skin infection (1 case) and subcutaneous hematoma (1 case). In the Group KP + IF, 11 patients (11/31) had complications including asymptomatic cement leakage (5 cases: 1 case into the intervertebral space, 2 cases lateral to the vertebral body and 2 cases into spinal canal), cerebral fluid leakage (1 case), subcutaneous hematoma (2 cases) and skin infection (3 cases).

Multiple comparisons using the Fisher’s exact test showed that the Group KP + IF had a higher incidence of postoperative complications than Group PKP (*P* = 0.0212). The complication rate of the Group PKP and Group IF was of no significant difference (*P* > 0.9999, Table 2).

## Discussion

MVF with posterior wall damage and no neurological deficit is becoming more common, for the cancer patients have longer survival and more often undergo imaging when there are symptoms of pain before the onset of neurological symptoms.

In this entity, conservative treatments are rarely effective, and surgical treatments are usually advocated. However, controversy remains regarding the selection of surgical procedures, which commonly include PKP, internal fixation, and kyphoplasty combined with internal fixation.

Compared with open surgery, PKP is a better surgical option in most cases of MVF for its minimally invasive nature. However, in MVF with posterior wall damage and no neurological deficit, kyphoplasty is considered relatively or even absolutely contraindicated [6–9], over safety concerns balloon inflation may push tumor into spinal canal, causing neurological injury [14]. Therefore, we preferred to perform open surgeries including internal fixation alone and internal fixation combined with KP of the affected vertebra at the early stage of this study. The satisfactory results of PKP in the treatment of osteoporotic vertebral fractures with posterior wall damage encourage us to perform PKP in the cases of MVF [10, 15]. In the present study, we did not observe any radiological findings of posterior vertebral body wall retropulsion during operation in Group PKP and Group KP + IF, and no resultant neurological injuries were caused.

The primary goal of surgical treatment was to address the pain. The pain caused by MVF can be divided into 2 types: local pain and mechanical pain. Local pain results from periosteal stretching and inflammation caused by tumor growth, and mechanical pain is ascribed to instability. In our study, all 3 groups gained significant pain relief after surgery. However, the analgesic effect differed among the three groups. At postoperative 3 days, Group PKP showed better pain reduction than Group KP + IF (*P* < 0.05). At other follow-up time points, there were no significant differences between Group PKP and Group KP + IF (*P* > 0.05). The difference at postoperative 3 days could be attributed to the highly invasive nature of KP + IF and the minimally invasive nature of PKP. At all follow-up time points, Group PKP and Group KP + IF showed greater pain relief than Group IF (*P* < 0.05). The results could be attributed to the use of PMMA cement which confers several advantages as follows: (1) PMMA cement augmentation can provide mechanical stabilization and sacrifice the sensory nerve ending, addressing local pain and mechanical pain in MVF. (2) PMMA cement has hyperthermia and cytotoxic effects, which can kill tumor cells and destroy tumor-feeding arteries, achieving local tumor control.

Stabilization of vertebral body to prevent further collapse and neurological injury is another goal of surgical treatment for MVF with posterior wall damage. Whether a single PKP procedure can provide long-term stability is invalidated. In our study, significant correction of LKA and restoration of the vertebral height were observed and were not lost during the follow-up period in all 3 groups. Although Group IF and Group KP + IF were more efficacious in LKA correction and vertebral height restoration than Group PKP, we found pain reduction was not correlated with correction of local kyphosis and vertebral height, which was consistent with previous studies [11, 16].

Cement leakage is the most common complication in the procedure of kyphoplasty, either percutaneous or open. For MVF with posterior wall damage, the risk of cement leakage is very high. Molloy et al. [11] reported that the cement leakage rate was 31% using PKP for the treatment of cancer-related vertebral fracture with posterior vertebral wall defect. Even higher rate up to 47.8% has been reported [17]. In the present study, the cement leakage rate was X in Group PKP and Y in Group KP + IF, which is comparably lower. In our experience, some leakage can be avoided with good techniques, such as cement injection under conditions of high viscosity and low pressure, continuous fluoroscopic monitoring, graded infusion technique and incremental temperature cement delivery technique. [13]

## Conclusions

The present study showed that PKP was safe and effective in treating MVF with posterior wall damage and no neurological deficit. It can achieve similar clinical outcomes compared to IF combined with KP, but associated with less operative time, less blood loss and fewer complications. IF alone should not be the first treatment option for its poorer analgesic effect.

## Data Availability

The datasets used and/or analyzed during the current study are available from the corresponding author on reasonable request.

## Abbreviations

AVH: Anterior vertebral heights
CT: Computerized tomography
IF: Internal fixation
KP: Kyphoplasty
LKA: Local kyphotic angle
MRI: Magnetic resonance imaging
MVF: Metastatic vertebral fracture
MVH: Middle vertebral heights
ODI: Oswestry Disability Index
PKP: Percutaneous kyphoplasty
PMMA: Polymethylmethacrylate
VAS: Visual analog scale
